# What do young people with rheumatic conditions in the UK think about research involvement? A qualitative study

**DOI:** 10.1186/s12969-018-0251-z

**Published:** 2018-05-24

**Authors:** Suzanne Parsons, Wendy Thomson, Katharine Cresswell, Bella Starling, Janet E. McDonagh, on behalf of the Barbara Ansell National Network for Adolescent Rheumatology (BANNAR)

**Affiliations:** 1 Public Programmes Team, Manchester University NHS Foundation Trust, Manchester, UK; 2 NIHR Manchester Biomedical Research Centre, Manchester University NHS Foundation Trust, Manchester, UK; 3 Manchester Academic Health Science Centre, Manchester, UK; 4 Arthritis Research UK Centre for Genetics and Genomics, Centre for Musculoskeletal Research, Division of Musculoskeletal and Dermatological Sciences, Manchester, UK; 5 School of Biological Sciences, Faculty of Biology, Medicine and Health, The University of Manchester, Manchester, UK; 6 Research and Innovation Division, Manchester University NHS Foundation Trust, Manchester, UK; 7 Arthritis Research UK Centre for Epidemiology, Centre for Musculoskeletal Research, Division of Musculoskeletal and Dermatological Sciences, Manchester, UK; 8 Centre for MSK Research, University of Manchester, Stopford Building 2nd floor, Oxford Rd, Manchester, M13 9PT UK

**Keywords:** Qualitative, Young people, Adolescent rheumatology, Rheumatic conditions, Patient involvement

## Abstract

**Background:**

Involving people of all ages in health research is now widely advocated. To date, no studies have explored whether and how young people with chronic rheumatic conditions want to be involved in influencing health research. This study aimed to explore amongst young people with rheumatic conditions, 1) their experiences of research participation and involvement 2) their beliefs about research involvement and 3) beliefs about how young people’s involvement should be organized in the future.

**Methods:**

Focus groups discussions with young people aged 11–24 years with rheumatic conditions across the UK. Data was analysed using a qualitative Framework approach.

**Results:**

Thirteen focus groups were held involving 63 participants (45 F: 18 M, mean age 16, range 10 to 24 years) across the UK. All believed that young people had a right to be involved in influencing research and to be consulted by researchers. However, experience of research involvement varied greatly. For many, the current project was the first time they had been involved. Amongst those with experience of research involvement, awareness of what they had been involved in and why was often low. Those who had previously participated in research appeared more positive and confident about influencing research in the future. However, all felt that there were limited opportunities for them to be both research participants and to get involved in research as public contributors.

**Conclusions:**

These findings suggest that there is an on-going need to both increase awareness of research involvement and participation of young people in rheumatology as well as amongst young people themselves.

## Background

Involving people at all stages of health research (including young people) is now widely recommended [1]. In the UK, many grant and all ethics application processes now ensure that patient and public involvement (PPI) in research is embedded within them [2]. PPI is also advocated by professional bodies in general and specifically for young people. [3, 4].

Within this study we defined involvement as “where members of the public are actively involved in research projects and in research organisations” [5] and participation as, “where patients and the public act as research participants”.

Involving patients and the public in research helps ensure that research is designed around their needs and what is important to them [6]. However, it can be challenging to involve a diverse range of people [7]. This is particularly true with young people [8]. In view of the major sociocultural influences on adolescent health [9], representative sampling (in terms of socio-demographics- gender, ethnicity, culture and urban vs rural regional variations) is vital in studies of adolescents in general and when not feasible, the limitations of non-representative samples should be stated. In view of the wide variation of normal puberty, chronological age is a poor indicator of developmental status. Therefore, attention to the stages of adolescent development is required in any research involving young people. Ensuring such diversity may also result in part from a lack of confidence and/or inexperience amongst researchers regarding PPI [10], particularly with respect to young people in these particular developmental stages as although clinical research focusing on young people is rapidly evolving, it is still developing compared to research with adult populations [11].

The Barbara Ansell National Network for Adolescent Rheumatology (BANNAR) is a network of rheumatology professionals aiming to ensure that young people in the UK have the best chance to benefit from developments in the field of adolescent and young adult rheumatology [12]. A key priority for BANNAR is to involve young people in developing the network’s research priorities.

This study aimed to explore (i) experiences of research participation and involvement (ii) beliefs about research involvement amongst young people with rheumatic conditions and (iii) beliefs about how young people’s involvement should be organized in the future.

Data regarding their research priorities has been published elsewhere [13].

## Methods

This was a qualitative study of young people with rheumatic conditions. Sixteen focus groups were planned (8 with 11–15 year olds and 8 with 16–24 year olds) in all four nations of the UK to capture the potential impact of differences in health service organisation on young people’s experiences. The age ranges were chosen to reflect adolescent developmental stages i.e. early and mid-adolescence (11–15 years) and late adolescence and young adulthood (16–24 years).. Sixteen focus groups were conducted in all four nations of the UK to capture the potential impact of differences in health service organisation on young people’s experiences. The methods for this project have already been reported in detail elsewhere [13, 14].

### Recruitment

Rheumatology team members gave study information sheets to a broad range of eligible young people (in terms of age, gender, ethnicity, condition, research experience and socio-economic status). Inclusion criteria were English speaking 11–24 year olds, under the care of a rheumatologist with any chronic rheumatic condition.

Young people were also recruited via a UK based charity, Arthritis Care to ensure that young people who were not under the care of rheumatologists associated with BANNAR were involved [15]. As the aim of the project was to obtain the views from young people with any chronic rheumatic condition ie not specific to any particular disease, the only demographic details collected on individual participants were gender and age.

Focus groups were moderated by SP (a social scientist) and/or JMcD (a Paediatric Rheumatologist) who had no direct involvement in the clinical care of participants.

### Focus group topic guide

The topic guide is described in detail elsewhere [14]. Focus groups lasted for up to 90 min and explored:

1. Experiences of research participation and involvement
2. Beliefs about the research process and young people’s involvement in it
3. Beliefs about how young people’s involvement should be organised in the future

### Data management

Focus group recordings were transcribed verbatim, and pseudonyms were created for names, organisations and places.

The data was analysed thematically, using the Framework approach to qualitative data analysis [16]. Framework allowed both apriori and emergent themes to be included within the analysis. The study topic guide was used as a starting point for the thematic framework, and then SP, KC and JM read through the transcripts and identified recurrent themes to further develop the framework. This framework was then applied to the data and further refined where necessary.

## Results

Thirteen focus groups were held across the UK (England 8; Scotland 2; Northern Ireland 2; Wales 1). The original aim had been to conduct 16 focus groups but data saturation was achieved after 11. We determined that no new ideas were generated via reviewing recordings and transcripts and early analyses of the data. We however conducted a further 2 focus groups to ensure that young people from all four nations of the UK had the opportunity to participate although no new ideas were identified in this groups.

Six groups were held with 11–15 year olds (*n* = 30) and seven with 16–24 year olds (*n* = 33). Participants’ ages ranged from 11 to 24 years (mean = 16), 20 of whom were male and 43 female. Characteristics of participants are detailed in Table 1.

**Table 1:** Characteristics of participants [13]

	UK	England	Scotland	Northern Ireland	Wales
Age group					
11–15 year olds	30	20	5	5	0
16–24 year olds	33	19	2	9	3
Gender					
Male	20	15	2	3	0
Female	43	24	5	11	3

The themes which were identified are detailed in Table 2.

**Table 2:** Key themes identified with Framework analysis

• Young people’s experiences of research participation	
◦ Experience of research participation as children ◦ Feedback on research participation ◦ Altruism as a motivator for research participation	
• Beliefs about and experiences of young people being involved in research	
◦ Motivations for involvement ◦ Role of experience of condition in involvement ◦ Experience of involvement	
• Challenges to, and facilitators of, young people’s involvement	
◦ Being taken seriously by and listened to by researchers ◦ Access to research and researchers ◦ Clear roles and importance of co-production ◦ Flexibility in involvement approaches	
• Practical considerations when involving young people in research	
◦ Timing of involvement ◦ Convenience of activity ◦ Role of incentives ◦ Patient-led versus researcher-led involvement	

### Young people’s experiences of research participation

Young people’s experience and understanding of research varied greatly. For example, some knew that they had been a research participant but could not recall the details of the research (Table 3a). This was due, in part, to the challenges of recalling childhood experiences when older (Table 3b) Some explained that their younger selves had been happy to leave the responsibility for understanding the details of the research to their parents. (Table 3c).

**Table 3:** Experience of Research Participation

	Examples of quotes
3a. Experience of research in childhood - Degree of understanding regarding the purpose of research when a child	F: I am 18 and I think I did one about a year and a half ago, and there was someone in the Children’s Hospital that kept asking for my saliva yeah. It did become a running joke between me and my dad that she just wants my germs! So yeah, I think I have (England over 16)
3b. Experience of research in childhood - Challenges of recalling research participation in childhood	F: Yes I have when I was younger, I can’t quite remember all the details, I think it was research in how metal joints affect the blood and that and when they test blood, they try and see whether or not they can judge how far…how worn out the joint is (Wales 16 and over)
3c. Experience of research in childhood - Parents taking control of research consent process when younger	F - The X study was okay because I was young enough that my mum came and kind of took control. (Northern Ireland 16 and over)
3d. Lack of feedback on research participation	F - The University one was slightly different. It was quite daunting, I had a one-to-one interview. Mine lasted just over three hours. But I could see the benefits of why I actually should do it, but I still haven’t actually received anything back from it. Sometimes you need to see what’s come off your participation. (Northern Ireland 16 and over)
3e. Lack of feedback on research participation	F: it is like taking a test and never getting your grade (Northern Ireland under 16)
3f. Lack of feedback on research participation	F – But I think it would be useful if people who have it know like what’s been found so they kind of like have got an idea of where it is all going (England under 16)
3g. Importance of feeling that your contribution to research is meaningful	M- I think it’s a trade-off between convenience and impact, if it’s going to really be helpful or if it’s really going to have a big impact. I feel that if I am just going to be a data point then it really doesn’t impress me that it is important. It was conveyed to me that it was a really important factor in their research, I think I’d put a lot more effort into it (England over 16)
3h. Reasons for research participation - Altruism	Facilitator – Why did you decide to take part? M- Well I’ve got it may as well help other people who have it. (England over 16)
3i. Reasons for research participation – Altruism	F- I kind of decided, because it took me probably three or four years to get diagnosed, I was thinking whether if it was easier for someone else to just be diagnosed straight away, So that’s kind of why I helped a bit. (England under 16)
3j. Reasons for research participation – Altruism	M- Not being rude but I think you are stupid if you don’t take part in research. If you’re upset about something and you want to get better, surely you would take part in something that might make you better in the long run and help others (England under 16)
3k. Reasons for research participation – Altruism – wanting to make things better	F: Exactly the reason I agreed to take part in this is so that young people won’t be in the same situation I was when I was diagnosed. (Wales over 16)

*F* Female *M* Male (country, age group)

Some participants however had clear memories of the research with a number expressing concern about the lack of feedback they had received on findings (Table 3d, e and f). Others spoke about the importance of clearly understanding what they were contributing to the research (Table 3g).

For some young people, altruism was a key reason for their participation, as they believed that taking part was the ‘right thing to do’ and that it would be impossible for young people in their position in the future to be helped if they did not participate (Table 3h, i, j and k).

Finally, a considerable number of participants reported being interested in research participation but could not recall ever being asked to take part in clinical research until the reported study.

#### Beliefs about and experiences of, young people being involved in research

Young people believed that they offered a valuable, different perspective on the research process compared to adults (including researchers) (Table 4a and c). They felt that contributing the lived experience of their condition to the research process was valuable and essential, as many had significant experience of their illness and its treatment since disease onset in early childhood (Table 4c, d, e and f).

**Table 4:** Beliefs about and experiences of young people being involved in research

Theme	Example quotes
4a. Motivations for involvement – young people’s views and experience are different than adults	F- I would do it because adults and people our age they think differently and they don’t always consider the things that we consider and because we’re the ones getting involved I think it’s good that we have a say. (England under 16)
4b. Motivations for involvement - altruism	M- If someone came to me I’d instantly say, yeah, I’ll take part in it. I don’t care if it’s a bit boring because it’s for the benefit of others. So there needs to be like a Facebook page or something, you know like an online poll and stuff like that (England over 16)
4c.Experience as a young person – Providing a different perspective to researchers	M- I disagree because they should give ideas on how the research is done, because it’s your opinion and if you’re going through something you should have a say on what the researchers are actually focusing on, because it’s happening to you. It would be useless if they were focusing on something that isn’t important to you. (England under 16)
4d. Experience of condition- Importance and value of young people’s experiences of their condition	F: Researchers think they are the best but sometimes they are not, young people have the problems in their body and they know more things about their condition than researchers do. (Northern Ireland under 16)
4e. Experience of condition - Importance and value of young people’s experiences of their condition	M: I think also it is quite important that we have experience. We have experience in the service, we have experience with the doctors and we have kind of had all or quite a substantial part of our life in the service so we know what it is like and we have had good experiences and bad experiences. (England 16 and over)
4f. Experience of condition - Importance and value of young people’s experiences of their condition to research	M: it is like a better thing as well, as you have the doctors who know about the thing, they know about condition and how to treat it but they don’t know what it is like to cope with something like that. (Scotland 16 and over)
4g. Experiences of involvement - Involved but unclear what in	F – He’s called M, he’s 17 and he’s been involved M – I think I have F – You’ve come on advisory groups for disease specific JIA and I’m sure there’s questionnaires and stuff. You’ve done involvement and the research side (England over 16)
4h. Experiences of involvement – involved but in a limited way	Facilitator - the other question was whether you’ve ever given your thoughts on how research is done M- Yeah, once or twice, but that’s only speaking to a researcher at the hospital, that just comes and talks to me (England under 16)
4i. Experiences of involvement – uncertain about whether played a useful role in advisory groups –	Two or three of you have said that you have taken part in advisory groups- have you had the chance to really have a say? F- I had a big rant one time, I don’t know, it was like one of those next door. I went so off topic and just ranted about the NHS really. F- it’s addressing things like mental health aspects, which might be more personal than information from blood samples (England over 16)
4j. Experiences of involvement – uncertainty on how or whether to input	M- I never talk during the advisory group thing … I just had nothing to say basically, They were saying things that I was thinking of saying but didn’t end up saying because they got there first. (England over 16)
4k. Experiences of involvement - challenges to participating in advisory groups	F- The last one I think was one of the better ones, because as I said there was less people there, but also because it was kind of like, sometimes in the bigger groups you can hear someone say something that you disagree with, but it’s a bit awkward because you don’t know them well enough to disagree with them, because you just don’t want to get into an argument. But that one, because we were basically all the same age and had the exact same thing, it was better. (England over 16)

*F* Female *M* Male (country, age group)

Experience of research involvement varied from considerable to no experience (Table 4g, h, i, j and k). Several reported prior involvement in advisory groups although they varied in their perceptions of the value of their contribution to such groups (Table 4h, I, j and k). As with research participation, altruism was a key driver for young people to become involved in shaping research (Table 4b ).

##### Challenges to and facilitators of, young people’s involvement

All participants were able to discuss their beliefs on the best approaches to involving young people in research (Table 5a-f) even if they had no prior experience. They stressed the importance of co-production of research with researchers and believed that involvement should be driven and organised by public contributors when possible (Table 5d). Participants advocated using both online and face to face approaches to involvement in addition to gaining a greater understanding of young people’s networks to facilitate their involvement (Table 5a, b and c). The use of social media was believed to be a key approach to facilitate the involvement of a wide range of young people in research (Table 5a).

**Table 5:** Challenges and facilitators to young people’s involvement

5a. Flexible approaches to involvement – role of social media and online approaches	F-. So I mean you’ve Facebook, you’ve twitter, which I’m pretty sure at least everyone in this room has at least one. Even the younger group. There would be some way of getting in contact with them in that manner. And it’s a free way of getting in contact with them; it can be monitored as well. And its instant results. And it means that you are not going to have the idea that you can’t go along as you can’t meet this person’s schedule, not a problem, use that. (Northern Ireland over 16)
5b. Flexible approaches to involvement – role of social media and online approaches	F: Maybe consulting more groups like us, we can maybe video conference if you wanted to give it a group discussion about something and the same for all different regions. (Wales over 16)
5c. Continued importance of face to face involvement	M- Yeah I like face to face, because it’s good to learn about other people, (England under 16)
5d. Clear roles and importance of co-production of research	M- I think both people should contribute and agree with what’s going on... it shouldn’t be just the doctors decision, it should be the patients’ as well. (England over 16)
5e. Access to research and researchers	M: I think there is I don’t know what the word is a barrier maybe between, you know, young people and researchers. And how you contact each other and even if we did sort of have to, how would we kind of work together? And put that into research. (Scotland over 16)
5f. Being taken seriously by researchers and feeling listened to	F- I think they have to take people who are actually suffering with it seriously, and how they are feeling it like, and they have to have some consideration for what they are doing. And to think whether it will actually benefit them in some way what they are actually researching into. (England over 16)

*F* Female *M* Male (country, age group)

Despite feeling that young people had a right to be involved, participants expressed uncertainty over the mechanisms by which young people could be involved in research and how this can best be supported (Table 4e). All believed that accessing information about research and research findings was challenging to both research participation and involvement (Table 5e).

##### Practical considerations when involving young people in research

Timing of involvement opportunities was a key issue, with young people discussing the importance of their personal commitments being considered when involvement requests were made (Table 6a, b and c). They also discussed the importance of researchers not asking recently diagnosed young people to be involved as they felt that at this time, young people would have too much to do to become familiar with their condition before they could consider research involvement (Table 6c). A further suggestion was for researchers to combine involvement with other activities young people are already doing (Table 6d).

**Table 6:** Practical considerations when involving young people in research

Theme	Example quotes
6a.Timing of involvement	M- I would probably say week days, because obviously people have like college or school or work or whatever. So like today (Saturday) there was no problem for me to come here (England over 16)
6b.Timing of involvement	F: Probably during holidays and all that, I have quite heavy workload with college and I have exams coming up and stuff so probably holiday kind of times. (Scotland over 16)
6c.Timing of involvement activity	F- I think as soon as you’ve been diagnosed, I think is probably the worst time, because I don’t think you know the disease yourself. So I think if like in the future people are getting diagnosed, I think they should wait a while until they’re familiar with their own disease before they start research. (England under 16)
6d.Convenience - Combining involvement with other things young people are doing	M- The most important thing about why you get involved with research is convenience, so try and maybe do what they did, because if I was to be asked to do this separately once every couple of months, I would probably think that this is going to be hassle. So rather than setting up a separate group, try and maybe go along to some other groups, like that lupus group or other support groups (England over 16)
6e.Incentives for involvement	M- People are going to be more receptive if they think there is a reward at the end. Because you could put out notices saying we need people to come and help and you’ll only get those people who are actively involved or who actively seek out these type of opportunities. But if you set some sort of incentive, you may get people who think it’s not the normal thing they do but they’re willing to help out. (Northern Ireland over 16)
6f.Patient led or researcher led involvement	M: I think that it would be better if someone was there for ad/medical stuff as and when needed, but we were allowed to just get on with the thing. Like you might need someone to give stimulus and ask a couple of questions. But generally I think it should be like it is today, with just us talking, just the patients. (England 16 and over)
6g. Patient led or researcher led involvement	F: So it is interesting the continuity and management of it and stuff, so you set that up so it is a patient led thing and that is different to having a researcher-led thing, as researchers can’t do a lot of obviously, it is time consuming… (England 16 and over)

*F* Female *M* Male (country, age group)

Young people discussed how offering compensation could influence the types of people who became involved. Some felt that offering compensation could lead to people becoming involved who did not feel strongly about the research, and others that it would facilitate a wider range of people considering involvement (Table 6e).

Young people also had views regarding how involvement should be organised with some specifically advocating facilitation but that this ideally should be “patient-led” (Table 6f and g).

## Discussion

To the best of our knowledge, this is the first study, to explore the experiences and beliefs of young people with rheumatic conditions about research involvement. Although young people felt that they had a right to be involved in research, experience of research involvement varied. This may reflect geographical variation in the research culture, i.e. the extent to which research participation and involvement of people in research were viewed as priorities and key elements of usual care by clinicians, researchers and their host institutions.

Young people also expressed a wish to increase their research awareness. This may have arisen in part due to difficulties in remembering whether they had taken part in research especially if what was involved was similar to usual care e.g. completing the Childhood Assessment Questionnaire [17].

If young people believe that they have a low awareness of research in general, then it is also likely to be difficult to increase their involvement as a public contributor. In this study some young people reported never being approached about research participation nor research involvement. This concurs with the findings of a previous UK audit of paediatric and adolescent rheumatology services which reported that at least 60% of centres did not involve young people in research other than as research participants [14].

An interesting finding was that some young people who had actually been involved in shaping research did not always perceive this as involvement. One explanation for this may be the lack of feedback they received following their involvement, or it may reflect lack of clarity of their role during the participation and involvement processes.

A key strength of this study was the inclusion of 63 young people from a broad range of ages and from all four UK nations, thereby reflecting a range of service provision and research opportunities. The variation in the experiences of research involvement and participation was an important finding, validating our approach and highlighting the need to conduct a national study. However, recruitment for this study was poor in some areas particularly if there was not a PPI or transition coordinator who could help with recruitment. In the development phase of this research, a survey of the 25 main paediatric rheumatology units identified that just five units had a team member with PPI included in their job description [14]. Furthermore, in order to limit recruitment bias, we adopted a maximum variety purposive sampling approach to sampling for this study. This aimed to encompass the range of AYA development by having focus groups for the first 2 stages (early and mid) and the latter 2 stages (late adolescence and young adulthood). We also involved centres in all 4 devolved nations of the UK in order to capture the range of services for this age group, in both urban and rural settings. There has been a significant growth in specialist services for this age group in recent years in the UK although significant delays in referral persist for certain conditions eg juvenile idiopathic arthritis [18]. To further maximise the diversity within our recruitment we recruited via charities in an attempt to recruit young people not being treated at centres with specific paediatric, adolescent and/or young adult rheumatology services. These young people potentially had less access to research opportunities.

However despite efforts to recruit a maximum variety sample, due to the relative small sample sizes required within qualitative research there will still be some limitations in terms of the diversity of the sample. For example, research-naïve young people or those with very mild disease and/or who are happy with their current care may not have perceived a study focused on beliefs about research involvement as being relevant to them. However we did successfully recruit such young people in this study. Another group less likely to engage in research are those young people who have become disengaged from their rheumatology care. Understanding their perspectives would be particularly valuable. Reasons for non-response are important in any research but particularly pertinent to adolescent health as some studies have shown that those who fail to respond have poorer outcomes compared to adult non-responders [19].

An important impact of this study is that, incorporating the clear advice regarding involvement in the current study, we have since established a national young person’s advisory group to inform BANNAR research.

The group is called Your Rheum (https://yourrheum.org/) [20] and is currently holding face to face meetings several times a year and involving young people online in research.

Finally, whilst acknowledging the exploratory nature of the current study, one could argue that many of these findings are also true for adult populations. However, implementing change in the adolescent and young adult group in relation to involvement, just as in clinical care, requires developmentally appropriate approaches which change over time as young people grow and develop [11].

Young people identified a lack of opportunity or a perception of poor access to research and research involvement as a primary barrier to research involvement. This suggests that efforts are needed to increase researchers’ awareness and understanding of PPI and its’ likely impact. Increased awareness may increase researchers’ confidence in involving young people in their work and lead to a wider range of research involvement opportunities becoming available. As part of this current project, models of good research involvement practice in this area beyond rheumatology were collated to serve as a future resource for researchers [21]. Since the Clinical Studies Group in Paediatric Rheumatology was established [22], research involvement outside of large teaching hospitals has significantly improved but further work is still required, if we consider the views of participants in the current study of their poor access to involvement opportunities. Investment into appropriate staffing for such initiatives is supported by the finding that recruitment to this particular study was better in those centres with a team member with PPI as part of their job description.

The findings from this exploratory study also suggest that further work is needed to increase young people’s awareness of rheumatology research within the UK. The national paediatric rheumatology clinical studies group, which supports a portfolio of clinical studies across the UK states:

*“All children and young people in the UK with a rheumatological condition may be given the opportunity to be enrolled in a clinical trial or well conducted clinical study from point of diagnosis onwards”* [22]*.*

The current study has revealed that it will be important to ensure both that the aims and purposes of young people’s involvement in research are made clear to them as well as receiving feedback on both their involvement as well as their research participation. It will also be important to explore the language used to explain research participation and involvement to young people to gain some insights into why the nature of involvement is sometimes misconstrued.

In this current study, few young people had experience of being involved in influencing research, with those treated in large teaching hospitals being more likely to report having experience. Despite this finding, all young people strongly believed that they should be involved in research, particularly as they had lived experience of their condition and could provide a perspective which would otherwise not be available. Acknowledgement of the lived experience of young people with rheumatic disease is therefore as imperative for researchers as has also been reported for clinicians [23].

Understanding and evaluating the impact of patient and public involvement in general, and for young people is becoming an increasingly important issue. Evaluation frameworks for involvement have been developed for adults but it is still unclear whether they can be used effectively with young people [24]. The involvement of young people in research has been reported to have a positive impact on recruitment and retention [25]. However, Holland et al. cautions practitioners “against assuming that participatory research per se necessarily produces “better” research data, equalises power relations or enhances ethical integrity” [26]. Therefore, research is needed to explore how young people perceive their roles as active research partners in the context of chronic health conditions when involvement could potentially be an additional burden [27].

## Conclusions

This exploratory study highlights the importance of further enhancing the culture of research in the adolescent and young adult age group, to increase young people’s awareness of opportunities for both research participation and research involvement. In the UK, BANNAR and the YOURR project have made initial steps in doing so in rheumatology.

### What is known about this subject?

Involving people of all ages in health research is now widely advocated. To date, no studies have explored whether and how young people with rheumatic conditions want to be involved in influencing health research.

### What this study adds?

- This study highlights the need to increase the culture of research in some clinical specialities (including Rheumatology) to improve young people’s access to research participation and involvement opportunities
- Providing support and training to researchers to increase their confidence in involving young people in their work is also likely to increase the number of research involvement opportunities available.
- Being flexible in the range of approaches used to involve young people in research may increase the likelihood that a more diverse group of young people will become involved.

## Abbreviations

BANNAR: Barbara Ansell National Network for Adolescent Rheumatology.
BSPAR: British Society for Paediatric and Adolescent Rheumatology
CHAQ: Childhood Health Assessment Questionnaire
NHS: National Health Service
PPI: Patient and Public Involvement
YOURR: Young People’s Opinions Underpinning Rheumatology Research
